# Reliability and validity of the Thai version of the PHQ-9

**DOI:** 10.1186/1471-244X-8-46

**Published:** 2008-06-20

**Authors:** Manote Lotrakul, Sutida Sumrithe, Ratana Saipanish

**Affiliations:** 1 Department of Psychiatry, Faculty of Medicine, Ramathibodi Hospital, Mahidol University, Bangkok 10400, Thailand; 2 Department of Family Medicine, Faculty of Medicine, Ramathibodi Hospital, Mahidol University, Bangkok 10400, Thailand

## Abstract

**Background:**

Most depression screening tools in Thailand are lengthy. The long process makes them impractical for routine use in primary care. This study aims to examine the reliability and validity of a Thai version Patient Health Questionnaire (PHQ-9) as a screening tool for major depression in primary care patients.

**Methods:**

The English language PHQ-9 was translated into Thai. The process involved back-translation, cross-cultural adaptation, field testing of the pre-final version, as well as final adjustments. The PHQ-9 was then administered among 1,000 patients in family practice clinic. Of these 1,000 patients, 300 were further assessed by the Thai version of the Mini International Neuropsychiatric Interview (MINI) and the Thai version of the Hamilton Rating Scale for Depression (HAM-D). These tools served as gold-standards for diagnosing depression and for assessing symptom severity, respectively. In the assessment, reliability and validity analyses, and receiver operating characteristic curve analysis were performed.

**Results:**

Complete data were obtained from 924 participants and 279 interviewed respondents. The mean age of the participants was 45.0 years (SD = 14.3) and 73.7% of them were females. The mean PHQ-9 score was 4.93 (SD = 3.75). The Thai version of the PHQ-9 had satisfactory internal consistency (Cronbach's alpha = 0.79) and showed moderate convergent validity with the HAM-D (r = 0.56; P < 0.001). The categorical algorithm of the PHQ-9 had low sensitivity (0.53) but very high specificity (0.98) and positive likelihood ratio (27.37). Used as a continuous measure, the optimal cut-off score of PHQ-9 ≥ 9 revealed a sensitivity of 0.84, specificity of 0.77, positive predictive value (PPV) of 0.21, negative predictive value (NPV) of 0.99, and positive likelihood ratio of 3.71. The area under the curve (AUC) in this study was 0.89 (SD = 0.05, 95% CI 0.85 to 0.92).

**Conclusion:**

The Thai version of the PHQ-9 has acceptable psychometric properties for screening for major depression in general practice with a recommended cut-off score of nine or greater.

## Background

Depressive illness constitutes a significant proportion of all disabilities caused by mental disorders and has significant public health and economic costs [1]. During the past two decades, health care professionals have made major progress towards the treatment of depressive disorders. A few treatment approaches and guidelines have been developed and proven to be effective. The situation is different, however, in developing countries where there are insufficient resources for treating mental disorders. There has been little progress in improving treatment modalities [2]. In Thailand, most patients with mental disorders are often treated by general practitioners (GPs) because the number of psychiatrists in the country is still small. Moreover, people are reluctant to seek help from a psychiatrist, because of existing prejudices against psychiatric disorders. This has been reported in studies from several countries [3-5]. Thus, GPs in Thailand, as in many other countries, play an important role in taking care of patients with mental heath problems.

Depression is frequently unrecognized and undertreated in primary care, as it is often expressed in terms of somatic symptoms and anxiety rather than typical depressive symptoms [2,6]. The conditions are worse in developing countries where GPs are faced with high patient loads. A study in Thailand has shown that 72% of GPs see more than 50 patients a day, making it difficult for them to care for patients with mental illness effectively [7]. Because of this challenge, case-finding instruments may play a crucial role in assisting GPs in identifying depressive disorders in their patients.

During the last few years, a few depression screening questionnaires have been developed in the Thai language. These include the Health-Related Self-Report (HRSR) scale [8], the Thai Depression Inventory (TDI) [9] and the Center for Epidemiological Studies Depression Scale (CES-D) [10]. Despite their good sensitivity and fair to good specificity, these questionnaires are too time-consuming or too burdensome for routine use in primary care due to the large number of items on their lists. There is thus a need for a case finding questionnaire that is brief and easy to administer. Such a questionnaire can improve the recognition of depression in primary care.

Recently, the nine-item Patient Health Questionnaire (PHQ-9), which is derived from the Primary Care Evaluation of Mental Disorders (PRIME-MD), has been used as a reliable depression screening tool in primary care, with a demonstrated good sensitivity and specificity for depressive disorder. With only 9 items, the PHQ-9 is substantially shorter than most other depression screening measures. It comprises of 9 diagnostic symptom criteria upon which the diagnosis of DSM-IV major depressive disorder is based. With only 9 questions/items, the questionnaire has the potential to serve as a dual-purpose instrument that establishes depressive disorder diagnoses using a categorical algorithm as well as grades the depressive symptom severity [11]. The PHQ-9 has been translated into various languages. A number of studies on its validity and reliability as a diagnostic measure as well as its utility in assessing depression severity and in monitoring treatment response have been published [12-15]. However, till now, the PHQ-9 had not been translated and validated in Thai language. The purpose of this study was to assess the reliability and validity of a Thai version of the PHQ-9 for screening for major depressive disorder in general practice.

## Methods

### Subjects and procedure

This study project was approved by the Ethics Committee on Human Experimentation of the Faculty of Medicine Ramathibodi Hospital. The patients were recruited between October 2006 and February 2007 from the outpatient clinic of the department of family medicine, Ramathibodi Hospital, Bangkok. This clinic functions as a primary care clinic of the hospital.

Every fifth patients attending the outpatient clinic of the department was invited to participate in the study while they were waiting for consultation. Informed consents were obtained after the aims and the objectives of the study had been explained to the patients and they had agreed to participate in the study. The data were collected until 1,000 cases were obtained.

After completing the questionnaires, 300 of the patients were then assessed for their depressive illness by a research assistant. Convenience sampling was used to recruit the sample. The research assistant was a clinical psychologist who was trained to use the Thai version [16] of Mini International Neuropsychiatric Interview (MINI), and the Thai version [17] of Hamilton Rating Scale for Depression (HAM-D). She was unaware of the patients' PHQ-9 results, and interviewed the patients until a total of 300 responses were attained.

### Measures

The Thai version of the PHQ-9 was translated from the original PHQ-9 [11]. We used the Thai version of the Mini International Neuropsychiatric Interview (MINI) [16] as the criterion standard for identifying the presence of major depressive disorder, and the Thai version of Hamilton Rating Scale for Depression (HAM-D) [17] as a gold-standard measure of symptom severity.

### Patient Health Questionnaire (PHQ-9)

The PHQ-9 is a self-report measure, consisting of 9 questions based on the 9 DSM-IV criteria for major depressive episode. It refers to symptoms experienced by the patients during the two weeks prior to answering the questionnaires. After obtaining permission from the copy right holder, the PHQ-9 was translated following the guidelines for cross-cultural adaptation of self-report measures [18]. The process included two independent forward translations of the original PHQ-9 into Thai, consensus between translators on a forward translation, back-translation by a bilingual English teacher, and a review of the back-translation. Ten patients attending the out-patient department were invited to complete and to give comments on the pre-final version. Final modifications and adjustments were made accordingly.

Unlike many other questionnaires, the original PHQ-9 uses simple statements without culture-specific phrases, so there were only a few problems in the translation. Among the problems we encountered was the translation of particular words or phrases such as 'feeling down', 'fidgety' and 'restless'. After some discussions, the investigators were able to find phrases in Thai that conveyed approximately the same meanings.

Scores for each item in the PHQ-9 range from 0 (not at all), to 1 (several days), 2 (more than half of the days) and 3 (nearly every day), while summed scores range from 0 to 27. The PHQ-9 can be used as a screening tool with recommended cut-off scores of ten or greater for the diagnosis of major depression [11]. It can also be used to establish a diagnosis following a categorical algorithm. A major depressive disorder is diagnosed if 5 or more of the 9 symptoms have been present at least more than half the days of the past 2 weeks and 1 of these symptoms has been either depressed mood or anhedonia.

### Mini International Neuropsychiatric Interview (MINI)

The MINI, version 5, is a standardized clinical diagnostic interview schedule for DSM-IV Axis-I disorders [19]. It can be reliably administered by lay interviewers who have appropriate training. The Thai version of MINI, which was translated from the MINI, version 5, was used in this study. In comparison with an interview done by a clinician, the Thai version of MINI showed a high sensitivity of 0.92 and specificity of 0.93 for the diagnosis of current major depressive episode [16]. The depression modules of the schedule were used in the study as the gold standard diagnostic tool.

### Hamilton Rating Scale for Depression (HAM-D)

The HAM-D is a well accepted research tool for measuring the severity of depression and the response to treatment [20]. The Thai version of the HAM-D has good internal consistency (alpha coefficients = 0.74). Its concurrent validity, as compared to the Global Assessment Scale, is also satisfactory (Spearman's correlation coefficient = -0.82) [17].

### Data analysis

The data obtained in this study was analyzed by the Statistical Package for the Social Science 10 (SPSS 10). The internal consistency of the PHQ-9 was measured by Cronbach's alpha coefficient. For validity analysis, we determined both criterion and convergent validity. Criterion validity tests a scale's performance in comparison to a gold standard. The Thai version of MINI, which is used for the diagnosis of major depressive disorder was used as a criterion standard. Convergent validity is present when a scale behaves according to hypothesized relationships between the two tests that presumably measure the same construct. In this study we tested the association between PHQ-9 and the HAM-D.

We determined the criterion validity of PHQ-9 by assessing the psychometric properties of various cut-off scores and of the categorical algorithm of the PHQ-9. The following test characteristics of the PHQ-9 compared with the MINI were calculated: sensitivity, specificity, predictive values and likelihood ratios. To determine the best cut-off score, the receiver operating characteristic (ROC) curve was constructed against the presence of major depressive disorder by the MINI. The area under the curve (AUC) was also calculated. To determine the convergent validity, Pearson's correlation coefficient was used to establish the relationship between the PHQ-9 and the HAM-D.

## Results

We excluded from the study 76 of the 1,000 respondents who participated in the study and 21 of the 300 respondents who were further assessed by the MINI and the HAM-D due to incomplete data. The remaining 924 (92.4%) and 279 (93%) cases were included in our analysis.

The mean age of the 924 participants was 45.0 years (SD = 14.3). There were 681 females (73.7%) and 243 males (26.3%). Sixty per cent of them were married and 37.7% of them had graduated from secondary school. The sociodemographic characteristics of the 279 respondents who were further interviewed were not different from the previous group.

The mean PHQ-9 score of the 924 subjects was 4.93 (SD = 3.75) with a range of 0 to 24. The median score was 4.0, with a skewness of 0.99 (SD = +0.08). Most of the participants (89.4%) had a low PHQ-score (score < 10). Only 1.9% scored 15 or higher (moderately severe depression) [11]. The mean PHQ-9 score of the 297 subjects who were assessed by the gold standard tools was 6.5 (SD = 4.29).

### Reliability and item analysis

Cronbach's alpha for the total scale was 0.79. The mean scores for all PHQ-9 items are shown in Table 1. Individual items of the PHQ-9 were scored on a scale of 0 to 3 for symptom severity. Two items that were endorsed most frequently were sleep problems and low energy. The item that was endorsed the least was suicidal ideation. All items, if deleted, would consistently decrease the total scale alpha, except item 9 (suicidal ideation) which also had the least item-total correlation (0.35).

**Table 1:** PHQ-9 item level values and item-total correlations (n = 924)

PHQ-9 item	Mean	SD	Corrected item-total correlation	Alpha if item deleted
1. Little interest or pleasure in doing things	0.67	0.66	0.54	0.76
2. Feeling down, depressed, or hopeless	0.61	0.69	0.61	0.75
3. Trouble falling or staying asleep, or sleeping too much	0.92	0.87	0.48	0.77
4. Feeling tired or having little energy	0.88	0.83	0.56	0.76
5. Poor appetite or overeating	0.67	0.78	0.45	0.78
6. Feeling bad about yourself – or that you are a failure	0.30	0.58	0.48	0.77
7. Trouble concentrating on things	0.48	0.68	0.45	0.77
8. Moving or speaking so slowly that other people could have noticed	0.30	0.55	0.45	0.78
9. Thoughts that you would be better off dead or of hurting yourself	0.09	0.34	0.35	0.79

### Validity analysis

The performance of the PHQ-9 against the diagnosis of major depressive disorder by the Thai version of the MINI as a criterion standard was examined. According to the MINI, 19 patients (6.8%) met the diagnosis of major depressive disorder. The mean PHQ-9 score for these patients was 13.21 (SD = 4.61) whereas the mean score for patients without the diagnosis of major depression was 6.01 (SD = 3.84).

The validity of the PHQ-9 as a diagnostic tool, using a categorical algorithm, had a sensitivity of 0.53, a specificity of 0.98, a positive predictive value (PPV) of 0.67, a negative predictive value (NPV) of 0.97, and a positive likelihood ratio of 27.37. It was suggested that the threshold for item 9 (suicidal ideation) be lower, from "more than half the days" to "several days", in order to raise the sensitivity of the questionnaire [11]. However, we found that this approach yielded poorer results. Although the sensitivity at the new threshold increased to 0.58, its specificity, positive predictive value (PPV), and positive likelihood ratio decreased to 0.96, 0.50 and 13.68, respectively.

**Figure 1:**
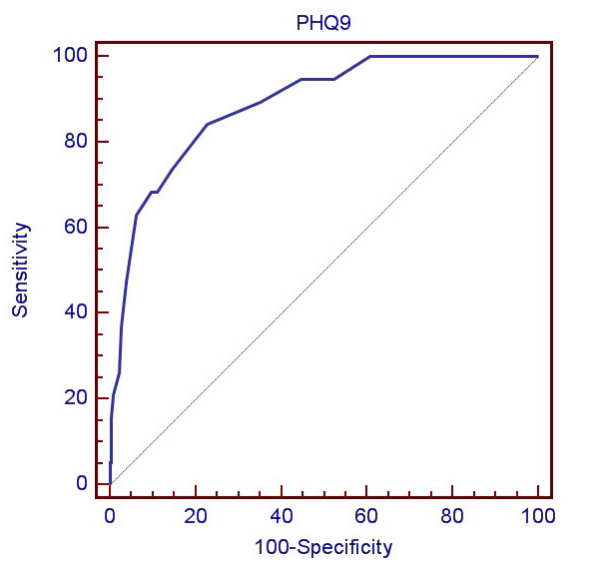
The Receiver Operating Characteristic (ROC) curve of the PHQ-9 versus the MINI for major depression diagnosis.

Table 2 demonstrates the sensitivity, specificity, PPV, NPV and likelihood ratio of different PHQ-9 thresholds in diagnosing major depression. At the cut-off score of the PHQ-9 of nine or greater, the sensitivity was 0.84 and the specificity was 0.77. This threshold had a PPV of 0.21 and a NPV of 0.99. The positive likelihood ratio was 3.71 at this cut-off point. The ROC curve illustrates that the PHQ-9 performed well in identifying patients with major depressive disorder (Fig 1). The area under the curve (AUC) in this study was 0.89 (SD = 0.05, 95% CI 0.85 to 0.92) which demonstrated a moderate accuracy [21].

**Table 2:** The performance of various PHQ-9 cut-off scores in detecting major depression

Cut-off score	Sensitivity	Specificity	Positive predictive value	Negative predictive value	Likelihood ratio – positive	Likelihood ratio – negative
≥ 6	0.95	0.48	0.12	0.99	1.81	0.11
≥ 7	0.95	0.55	0.13	0.99	2.12	0.095
≥ 8	0.89	0.65	0.16	0.99	2.56	0.16
≥ 9	0.84	0.77	0.21	0.99	3.71	0.2
≥ 10	0.74	0.85	0.27	0.98	5.04	0.31
≥ 11	0.68	0.89	0.31	0.97	6.13	0.36
≥ 12	0.68	0.90	0.34	0.98	7.12	0.35
≥ 13	0.63	0.94	0.43	0.97	10.26	0.39
≥ 14	0.47	0.96	0.47	0.96	12.32	0.55
≥ 15	0.37	0.97	0.50	0.95	13.68	0.65

To determine the convergent validity of the PHQ-9, the total score of PHQ-9 was correlated with the HAM-D. The Pearson's correlation coefficient between the PHQ-9 and the HAM-D was 0.56 (p < 0.001). This indicated a positive association of moderate strength between the two instruments.

We divided the HAM-D score into 4 groups according to the severity of depression [22]. We hypothesized that subjects with major depression would have higher PHQ-9 scores compared to subjects with mild depression or no depression. As shown in the Table 3, the group of patients with major depression as diagnosed by the HAM-D had a mean PHQ score of 14, followed by mean PHQ scores of 10.05 and 8.14 in patients with moderate depression and mild depression, respectively. These differences among the four PHQ-9 severity subscales were statistically significant (ANOVA: F = 31.91, df = 3, 275, p < 0.0001).

**Table 3:** Relationship between PHQ-9 mean scores and depression severity by HAM-D

HAM-D score [22]	N	Mean PHQ-9	SD	95% CI
No depression (0–7)	210	5.42	3.73	4.92–5.93
Mild depression (8–12)	36	8.14	2.98	7.13–9.15
Moderate depression (13–17)	20	10.05	3.87	8.24–11.86
Major depression (18 or greater)	13	14.00	4.93	11.02–16.98

## Discussion

The Thai version of PHQ-9 has several potential advantages. It is shorter than other diagnostic tools available in Thailand for identifying depression. It was primarily developed for use in primary care settings and is the only Thai questionnaire that has been tested in a primary care sample. With this instrument, both the presence of clinical depression and its degree of severity may be assessed. In primary care settings, the screening tool used for detecting the presence of depression alone may not be a reliable indicator of depression-related impairment. Instruments that can be used in both screening and scaling modes have a particular advantage in that their weaknesses can be compensated by each other [23].

The internal consistency of the PHQ-9 in this study (alpha coefficient = 0.79) was lower than in the studies from the United State (alpha coefficient = 0.79–0.89) [12,13]. However, its reliability was within the acceptable range. For a self-report instrument to be reliable, it is suggested that Cronbach's alpha be at least 0.70 [24]. The low mean PHQ-9 score of 4.9 in our study, compared to the mean scores of 6.0–6.5 in the US sample [12], may reflect a possible tendency in our patients to underreport symptoms via self-report questionnaire compared to the semi-structured interview.

The patients in this study had high rates of endorsement of somatic symptoms, i.e. sleep problems, low energy, and poor appetite. This is consistent with results from our previous study of depressed Thai patients, in which patients mostly emphasized somatic symptoms when they first reported their symptoms [25]. A recent study from four different racial groups in the United Stated also showed similar findings. Interestingly, the US subjects had a higher mean score in somatic symptoms such as sleep problems (0.96 to 1.37) and low energy (1.24 to 1.41) compared to our Thai samples (0.92 for sleep problems and 0.88 for low energy), whereas rates of endorsement of emotional symptoms such as anhedonia between our samples (0.67) and the US samples (0.56 to 0.89) were not very different. Our results support Kirmayer's argument [26] that subjects from countries that discourage expression of emotional distress do not report more somatic symptoms than subjects from countries where expression of emotional distress is accepted or even encouraged.

Result of the PHQ-9 categorical algorithm for detecting major depressive episodes showed that it provided a high specificity. However, its sensitivity was poor (0.53), rendering this algorithm less useful for screening purposes than the cut-off score. On the other hand, its high positive likelihood ratio (LR+) of 27.4 may make it a suitable method for a diagnostic purpose.

When the PHQ-9 was examined as a continuous measure, its validity was supported by the AUC value which suggests a moderate accuracy of the questionnaire. The sensitivity at the cut-off value of 9 or greater was 0.84 and the specificity was 0.77. These values are within an acceptable range. Sensitivity of screening instruments is considered good when their range is 0.79–0.97 and when their specificity is 0.63–0.86 [27]. The low specificity of the summed PHQ-9 score for diagnosing major depression is due to the fact that it is possible to diagnose the disorder without having either of the two cardinal symptoms of major depression. As such, the summed score does not match perfectly with the MINI, which is a structured diagnostic interview based on DSM-IV criteria [28].

The cut-off score at this point yielded the low PPV (0.21) and high NPV (0.99). Results form other studies of the PHQ-9 showed PPV ranging from 0.31 to 0.51 (depending on the cut-off) [11]. The PPV is the probability of disease if the patient's test is positive, while NPV indicates the probability that the patient is disease-free if the test is negative. A low PPV may indicate lower specificity, or a lower disease prevalence in the population undergoing screening, or a combination of these factors [29]. In this study, results may come from both low specificity (0.77) and low disease prevalence (6.8%).

We determined the convergent validity of the PHQ-9 in relation to the HAM-D. The satisfactory correlation between these two scales confirmed the validity of the PHQ-9. Recently, the PHQ-9 was also demonstrated to be sensitive to change and responsive to treatment outcomes over time and thus is a useful tool for monitoring treatment progress [14,30,31].

Our study had several limitations. First, the study was conducted in a university hospital; therefore, the respondents may not be representative of the actual primary care patients seen in a rural and remote area. That is, on average, the depressive patients in the family practice clinic of Ramathibodi Hospital are less severe than the depressive patients attending primary care in remote area. This is because when they or their relatives think that their symptoms are severe, they may go to the psychiatric out patients unit of Ramathibodi Hospital directly; whereas in a rural hospital, there is no psychiatric out-patient unit. In such a situation, all depressive patients have to attend the primary care unit of the hospital in the rural area, no matter how severe they are. For this reason, the respondents at the Ramathibodi Hospital may not exactly represent the actual primary care patients seen at hospitals in rural and remote areas. However, these patients with severe symptoms were only a small proportion of cases and our main objective of the study was to assess the ability of the tool to identify borderline cases of depression, which is a major problem in primary care settings. Second, there was a possibility that a proportion of participants might have underreported their depressive symptoms on both the PHQ-9 and the MINI clinical interview. Third, although the Thai version of the MINI performed well in a validity study, due to its highly structured instrument, it is still possible that it overestimated or underestimated the rate of depression in this study. Despite its potential shortcoming, however, the MINI was easy to understand because it was structured in simple, lay language. Thus few problems were encountered in the process of administering the instrument. This was also the case in the study of Adewuya et al [32]. Fourth, the test-retest reliability of the PHQ-9 was not assessed. Generally, this type of reliability is used for measuring the stability of a scale over time, and it is usually assessed after a short period of time. Unfortunately, most of the participants only had follow up appointments at the hospital at least one month after they first took the test. This time period is too long to assess the test-retest reliability.

Caution should be exercised that, although the PHQ-9 is a valid and useful self-rating instrument for screening for depression, routine usage of the questionnaire, in isolation, may not improve the quality of care as anticipated. Results from a review have revealed that the routine employment of screening questionnaires for depression alone has minimal impact on clinician's detection, management, and outcome of depression [33]. Moreover, in developing countries, new psychiatric cases identified by such instruments may be just an add-on burden to GPs whose workload is already heavy. Financial and institutional constraints in health care services should be taken into account before adopting such service delivery programs in order to maintain successful care [34]. In a region where there is a shortage of GPs, besides the benefit of a case detecting tool, a structured approach that facilitates an increased role of non-medical staff, patients, and family members may be more appropriate [35].

In summary, the Thai version of the PHQ-9 has acceptable psychometric properties for screening for major depression in general practice with a recommended cut-off score of nine or greater. Due to the low PPV in this study, further clinical assessment is recommended if a test result is positive. Because the categorical algorithm of the PHQ-9 yielded low sensitivity, it is less suitable for a screening purpose.

## Abbreviations

The Patient Health Questionnaire 9: PHQ-9; the Mini International Neuropsychiatric Interview: MINI; the Hamilton Rating Scale for Depression: HAM-D; positive predictive value: PPV; negative predictive value: NPV; area under the curve: AUC; receiver operating characteristic curve: ROC curve.

## Competing interests

The authors declare that they have no competing interests.

## Authors' contributions

ML conceived the study, designed the protocol, analyzed the data, and prepared the manuscript. SS participated in the study design, assisted in data collection, and gave significant comments on the manuscript. RS participated in the study design, coordinated among research assistants during the data collection process, and gave significant comments on the manuscript. All authors have read and approved the final version of the manuscript.

## Pre-publication history

The pre-publication history for this paper can be accessed here:


